# Chronic viral hepatitis in HIV infected patients

**DOI:** 10.1186/1471-2334-13-S1-P92

**Published:** 2013-12-16

**Authors:** Irina Niculescu, Augustin Cupşa, Florentina Dumitrescu, Andreea Stoian, Iulian Diaconescu, Manuela Muşa, Alina Crețu, Maria Bălan

**Affiliations:** 1 University of Medicine and Pharmacy, Craiova, Romania; 2 “Victor Babeş” Hospital for Infectious Diseases, Craiova, Romania

## Background

Chronic viral hepatitis, not an uncommon situation in clinical practice, raises problems when it comes to the evolution and management of the HIV co-infection. The objective of this paper was to evaluate the prevalence, etiology and evolution of chronic viral hepatitis infections among HIV infected patients (HIP).

## Methods

We performed an observational, retrospective study (1994-2010) on 826 HIP in the evidence of the Craiova HIV/AIDS Regional Centre; we analyzed the clinical and biological data regarding the viral hepatitis chronic infections among HIP.

## Results

Of the 826 HIP evaluated during the study period, chronic HBV infection was found in 198 HIP (23.97%), chronic HCV infection in 6 HIP (0.73%). Chronic HBV-HDV coinfection was detected in 6 HIP (9.37%, of 64 HIP evaluated) and chronic HBV-HCV coinfection in 2 HIP (0.24%). The evolution of chronic HBV infection during the study period was as follows: HBs seroconversion in 8 HIP (4.04%), chronic hepatitis-167 HIP (84.34%), cirrhosis-17 HIP (8.59%), undefined (lost to follow-up)-6 HIP (3.03%). Complete data necessary for the evaluation of HBV infection phase were available for 27 HIP; according to these data, the distribution of the evaluated subjects was as follows: HBe antigen negative chronic hepatitis B was detected in 8 HIP (29.63%), HBe antigen positive chronic hepatitis B in 6 HIP (22.22%) and immune control phase in 13 HIP (48.15%). 54 deaths (27.27%) were recorded among chronic HBV infected subjects; 18 deaths (9.10%) were liver related. The evolution of chronic HCV infection during the study period was as follows: chronic hepatitis in 3 HIP (50%) and cirrhosis in 3 HIP (50%). 1 death (16.67%) was recorded among chronic HCV infected subjects; this death was liver related.

## Conclusion

Chronic viral hepatitis infections are common among HIP, and chronic HBV infection occupies an important place. The evolution of chronic viral hepatitis infections raises problems through its increased morbidity and mortality.

